# Preliminary investigation into the genetic etiology of short stature in children through whole exon sequencing of the core family

**DOI:** 10.1515/biol-2022-0853

**Published:** 2024-05-08

**Authors:** Jinshui He, Shuyun Zhang, Yueya Kang, Yugui Zhang, Zhugui Zheng, Minyi Ruan

**Affiliations:** Department of Child Growth and Development, Zhangzhou Affiliated Hospital of Fujian Medical University, Zhangzhou 363000, Fujian, China; Department of Child Growth and Development, Zhangzhou Affiliated Hospital of Fujian Medical University, Zhangzhou 363000, Fujian, China; Department of Ophthalmology, Zhangzhou Affiliated Hospital of Fujian Medical University, Zhangzhou China

**Keywords:** short stature, genetic etiology, whole exon sequencing, gene mutations

## Abstract

A comprehensive survey was carried out to investigate the genetic etiology of short stature in children by whole exon sequencing of a core family cohort to find and study mutations in multiple genes to assess their potential correlations to low height in children. The study included 56 pediatric patients from the Department of Pediatrics at the Zhangzhou Affiliated Hospital of Fujian Medical University. The participants met strict inclusion criteria, including age, Han Chinese ethnicity, low height standard deviation score, and the absence of known causes for short stature. Core pedigrees were identified using exome sequencing. After sequencing, variations were categorized and interpreted according to a variety of factors, including inheritance, location, type, and disease-causing gene databases. Variants were verified by Sanger sequencing. Most of the 97 gene mutations were missense. ACAN, PHEX, and COL2A1 were the most common gene mutations. Copy number variations were identified, particularly associated with the PHEX gene. Protein functional studies revealed that the mutations had a considerable influence on disease-promoting damage. The chromosomal locations with the highest enrichment of these genes were chr12, chr5, and chr2. In conclusion, the study revealed numerous genetic changes that may substantially impact physiological processes and disease. These findings establish the basis for further investigations into their diagnostic and therapeutic capabilities.

## 1. Introduction

Short stature, defined as a height below the third percentile for a child’s age and gender, is a prevalent problem in pediatric medicine [1,2]. It affects about 3% of children and can have serious consequences for their physical and psychological health [3]. While some cases of short stature are attributed to environmental factors, such as malnutrition or chronic illness, a substantial proportion is believed to have a genetic basis [4–6].

In the broader domains of personalized medicine and genetics, it is critical to comprehend the genetic variations and their prospective effects on biological systems and human health. Genetic variations are of paramount importance in ascertaining an individual’s medication response, disease susceptibility, and overall health. The ability to recognize and analyze these variations yields vital knowledge regarding the genetic predispositions of an individual, thus facilitating personalized strategies for healthcare and treatment. In the context of genetics, the study of genetic variations is essential for comprehending the complexity of human genetic diversity. Millions of genetic variants comprise the human genome, and each of them has the potential to affect a variety of physiological characteristics, including height, disease susceptibility, and drug metabolism. An increased comprehension of the genetic basis of human diversity and susceptibility to various health conditions results from the investigation of these variations. Furthermore, in the field of personalized medicine, the analysis of genetic variations holds great promise for tailoring medical treatments and interventions to individual patients. Healthcare providers can enhance the efficacy and precision of medical care by providing personalized suggestions for disease prevention, diagnosis, and treatment through the identification of specific genetic variants associated with certain conditions.

Hence, it is imperative to comprehend the genetic basis of short stature to facilitate accurate diagnosis, prognosis, and potentially targeted therapeutic interventions [7,8]. Over the years, numerous genes have been implicated in the regulation of skeletal growth and development [9]. For example, genetic short stature can result from defects in the growth hormone-insulin-like growth factor axis (GHRH-GH-IGF-1) [10,11]. This pathway includes the production and release of growth hormone-releasing hormone (GHRH) from the hypothalamus, which stimulates the secretion of growth hormone (GH) from the pituitary gland [3, 12]. GH subsequently stimulates insulin-like growth factor 1 (IGF-1) production in the liver and other tissues, thus promoting overall and skeletal development [13,14].

Numerous genes have been associated with the GHRH-GH-IGF-1 axis; however, isolated growth hormone deficiency (IGHD) cases have most frequently reported mutations in GH1, GHRHR, and GHSR [4,15]. In addition, rare reports have described IGHD-causing mutations in BTK, SOX3, and HESX1 [11,16]. The diagnostic rate for IGHD varies between 6 and 11%, depending on the inclusion criteria and candidate genes used in different studies [7].

Short stature may also be a manifestation of multiple pituitary hormone deficiency (MPHD), which also is referred to as combined pituitary hormone deficiency (CPHD) [17,18]. MPHD/CPHD is associated with mutations in various genes such as HESX1, PROP1, POU1F1, PITX2, LHX3, LHX4, GLI2, GLI3, OTX2, SOX2, SOX3, FGF8, FGFR1, and IGSF1 [19–22]. Among these, PROP1 gene mutations are the most prevalent, resulting in deficiencies of GH, prolactin, thyroid-stimulating hormone, luteinizing hormone, and follicle-stimulating hormone, and sometimes also adrenocorticotropic hormone [23,24].

Additionally, certain short-statured individuals may be affected by growth hormone insensitivity (GHI), which is also referred to as Laron syndrome [20]. Gene mutations including those in GHR, STAT5B, PTPN11, IKBKB, IGF1, IGFALS, and insulin-like growth factor 1 receptor gene (IGF1R) can lead to GHI. Some patients who were previously diagnosed with idiopathic short stature were subsequently discovered to have GHI [18,19].

However, there is still a group of children that are suspected to have problems in the GHRH-GH-IGF-1 axis but do not show any known pathogenic mutations when screened for the above-mentioned genes [2,3]. This observation implies the existence of unidentified genes that play a role in this endocrine axis. Additionally, many of the functionally relevant genes have not been thoroughly investigated for pathogenic mutations. Hence, to clarify the cause of short stature in this population, it is imperative to conduct additional research and extensive investigations into this endocrine axis.

The primary aim of this study was to conduct a comprehensive investigation into the genetic factors contributing to short stature in children, in particular, to find and analyze genetic mutations through whole exon sequencing to ascertain whether they are associated with low height in pediatric patients; to address the knowledge gap regarding the genetic causes of short stature to pave the way for future developments in the field of diagnosis and treatment; to source a core family cohort; to perform whole exon sequencing; to classify and interpret variations; and to validate results using Sanger sequencing. The study was driven by the hypothesis that short stature is substantially influenced by genetic mutations and that whole exon sequencing can provide important insights into the genetic basis of this condition.

## 2. Materials and methods

### 2.1 Case source

This is a prospective observational study. The majority of patients included in this study were from the Department of Pediatrics at the Zhangzhou Affiliated Hospital of FuJian Medical University. These patients were referred by experienced clinicians who specialize in diagnosing and treating short stature. These clinicians did thorough clinical examinations, made initial diagnoses, gave treatment assistance, and conducted follow-ups.


**Informed consent:** Informed consent has been obtained from all individuals included in this study.
**Ethical approval:** The research related to human use has been complied with all the relevant national regulations, institutional policies and in accordance with the tenets of the Helsinki Declaration, and has been approved by the authors’ institutional review board or equivalent committee.

### 2.2 Inclusion and exclusion criteria

The criteria for inclusion were as follows: (1) minimum age requirement of 18 years; (2) Han Chinese ethnicity; (3) height standard deviation score for sex and age (HtSDS) not exceeding −2.5 (as per the standardized growth curve for Chinese children and adolescents, 2009); (4) comprehensive medical records, excluding patients with a clearly defined cause for short stature; (5) informed consent obtained from the patients and/or their guardians; and (6) collection of complete familial blood samples, including at least the patient and both parents’ EDTA anticoagulated blood samples.

Exclusion criteria were as follows: (1) birth trauma or asphyxia; (2) intracranial trauma, infection, or tumors; (3) chronic diseases affecting the digestive, renal, cardiac, or hematologic systems; (4) nutritional disorders; (5) other endocrine disorders (e.g., congenital hypothyroidism, congenital adrenal hyperplasia); (6) identifiable genetic metabolic disorders (e.g., mucopolysaccharidoses, glycogen storage diseases, methylmalonic acidemia); (7) chromosomal abnormalities (e.g., Turner syndrome); and (8) identifiable skeletal diseases (e.g., chondrodysplasia, hypophosphatemic rickets).

### 2.3 Patient recall

The procedures for patient recall were as follows: (1) obtaining informed permission forms; (2) collecting venous blood samples from enrolled patients and preserving them at −20°C following the extraction of genomic DNA using DNA extraction kits; and (3) collecting pre- and post-treatment clinical data for the enrolled patients and creating a clinical database using EXCEL software for archival and management purposes.

### 2.4 Whole exon sequencing technology to detect the families of enrolled children

Exome sequencing of the core pedigrees was performed using the Agilent SureSelectXT Library Prep Kit ILM reagent set for DNA library preparation, along with the Human All Exon V5 probes developed by Agilent. The specific procedures followed the standard SureSelect exome capture protocol, including genomic DNA fragmentation, end repair, adapter ligation, PCR amplification, probe hybridization to target regions, and enrichment using magnetic beads. Sequencing was carried out on Illumina HiSeq2500 or Next500 platforms, with a sequencing length of 2 × 125 bp.

Data processing involved the following steps: (1) after sequencing, raw data in FASTQ format were obtained from the sequencing platform. Using command-line tools in a Linux work environment, the data was processed as follows: (i) the BWA software (Burrows-Wheeler Alignment tool) compared the raw sequencing data with the human reference genome (hg19) to obtain BAM-formatted files, which could be visualized using the IGV software. (ii) The Picard software was used to remove duplicate reads and evaluate data quality. (iii) Single-nucleotide variations and insertions/deletions (Indels) were identified using the GATK software (Genome Analysis Toolkit), which produced files in VCF format. The wANNOVAR software (http://wannovar.usc.edu/) was then utilized to annotate the variants from various perspectives, facilitating subsequent filtering. Variant filtering mainly relied on mutation frequency, as well as the location and type of the variants. Databases such as the 1000 Genome Project, ESP6500, and ExAC (Exome Aggregation Consortium) were used as references for mutation frequency.

The analysis and interpretation of the results involved the following steps: rare variants that passed the frequency and type filtering criteria were evaluated based on whether they were inherited from parents and whether they were documented in OMIM (http://www.omim.org/) and HGMD (http://www.hgmd.org/) as disease-causing genes. Clinical presentations and inheritance patterns documented in previous reports, along with predictive analysis of the variants using tools such as PolyPhen-2 (http://genetics.bwh.harvard.edu/pph2), SIFT (http://sift.jcvi.org/), and Mutation Taster (http://www.mutationtaster.org/), were considered in a comprehensive assessment of the pathogenicity of the variants. The ACMG classification criteria for variants were consulted, and considering the experience from previous studies, rare variants that matched the clinical presentations and inheritance patterns were considered pathogenic if they fell into the following categories: previously reported pathogenic mutations and new mutations in known disease-causing genes (including nonsense, frameshift, start codon, stop codon, and splice-site mutations). Further investigation of novel missense variants, which have been identified as disease-causing genes and have been verified to be associated with height variation using GWAS, but have not been reported to have pathogenic mutations, would have scientific significance.

Primers were designed utilizing Primer Premier 5.0 software to validate the results. Sanger sequencing was then conducted to confirm the pathogenic or potentially pathogenic variants and determine their parental origin. If no pathogenic variants were found through exome sequencing, further genetic chip testing would be conducted.

## 3. Results

### 3.1 General information

This study included a total of 56 patients, consisting of 39 males (69.64%) and 17 females (30.36%). The mean age of the participants at the clinic presentation was 6.12 ± 3.14 years. For male and female patients, the mean genetic target heights were 165.98 ± 3.67 and 156.57 ± 4.36 cm, respectively. Regarding the HtSDS, our population presented a mean score of −3.29 ± 0.91, indicating an overall reduced stature compared to the standard population. Meanwhile, the mean peak GH value was found to be 8.63 ± 4.04.

**Table 1:** Possible pathogenic gene mutation and its variation type, related disease, and source of variation

Gene	Variation type	Related disease	Related disease hereditary mode	Source of variation
ABCC9	Heterozygosis type	OMIM: 619719	AR	Paternal heterozygosis
ACAN	Heterozygosis type	OMIM: 608361	AD	Paternal heterozygosis
AFF4	Heterozygosis type	OMIM: 616368	AD	Paternal heterozygosis
ALPL	Heterozygosis type	OMIM: 146300	AD/AR	Paternal heterozygosis
AMMECR1	Hemizygote type	OMIM: 300990	XLR	Non-paternal heterozygosis
ANKH	Heterozygosis type	OMIM: 118600	AD	Maternal heterozygosis
ARID1B	Heterozygosis type	OMIM: 135900	AD	Paternal heterozygosis
AUTS2	Heterozygosis type	OMIM: 615834	AD	Maternal heterozygosis
BMP2	Heterozygosis type	OMIM: 617877	AD	Paternal heterozygosis
CHD7	Heterozygosis type	OMIM: 214800	AD	Maternal heterozygosis
CLCN7	Heterozygosis type	OMIM: 166600	AD	Maternal heterozygosis
COL10A1	Heterozygosis type	OMIM: 156500	AD	Maternal heterozygosis
COL11A1	Heterozygosis type	OMIM: 228520	AR	Paternal heterozygosis
COL1A1	Heterozygosis type	OMIM: 619115	AD	Paternal heterozygosis
COL1A2	Heterozygosis type	OMIM: 259420	AD	Paternal heterozygosis
COL2A1	Heterozygosis type	OMIM: 200610	AD	Paternal heterozygosis
COL9A2	Heterozygosis type	OMIM: 614284	AR	Paternal heterozygosis
COMP	Heterozygosis type	OMIM: 132400	AD	Paternal heterozygosis
CREBBP	Heterozygosis type	OMIM: 618332	AD	Paternal heterozygosis
CSNK2A1	Heterozygosis type	Other related disease	AD	De novo variation
CUL7	Heterozygosis type	OMIM: 273750	AR	Paternal heterozygosis
DYM	Heterozygosis type	Other related disease	AR	Uninspected source
DYNC2H1	Heterozygosis type	OMIM: 613091	AR/DR	Paternal heterozygosis
DYSF	Heterozygosis type	Other related disease	AR	Uninspected source
EBP	Deletion type	OMIM: 302960	XLD	Maternal heterozygosis
EFTUD2	Heterozygosis type	OMIM: 610536	AD	Maternal heterozygosis
ENPP1	Heterozygosis type	OMIM: 208000	AR	Maternal heterozygosis
ERCC5	Heterozygosis type	OMIM: 278780	AR	Paternal heterozygosis
EVC2	Heterozygosis type	OMIM: 193530	AD	Maternal heterozygosis
FGF23	Heterozygosis type	OMIM: 193100	AD	Paternal heterozygosis
FGFR1	Heterozygosis type	OMIM: 147950	AD	Maternal heterozygosis
FGFR2	Heterozygosis type	OMIM: 101400	AD	Maternal heterozygosis
FGFR3	Heterozygosis type	OMIM: 100800	AD	Maternal heterozygosis
FLNA	Hemizygote type	OMIM: 304120	XLD	Maternal heterozygosis
FLNB	Heterozygosis type	OMIM: 150250	AD	Paternal heterozygosis
FN1	Heterozygosis type	OMIM: 184255	AD	Maternal heterozygosis
G6PD	Hemizygote type	OMIM: 300908	XLD	Uninspected source
GHRHR	Heterozygosis type	Other related disease	AR	Uninspected source
GHSR	Heterozygosis type	OMIM: 615925	AD/AR	Paternal heterozygosis
GJB6	Heterozygosis type	OMIM: 129500	AD	Paternal heterozygosis
GLI2	Heterozygosis type	Not reported	Not reported	Maternal heterozygosis
GNAS	Heterozygosis type	OMIM: 166350	AD	De novo variation
HESX1	Heterozygosis type	Other related disease	AD/AR	Non-maternal heterozygosis
HMGA2	Heterozygosis type	OMIM: 618908	AD	Maternal heterozygosis
HUWE1	Hemizygote type	OMIM: 309590	XL	Maternal heterozygosis
IFITM5	Heterozygosis type	OMIM: 610967	AD	Paternal heterozygosis
IGF1R	Heterozygosis type	OMIM: 270450	AD/AR	Non-paternal heterozygosis
IGF2	Heterozygosis type	Other related disease	AD	Paternal heterozygosis
IGFALS	Heterozygosis type	Other related disease	AR	Uninspected source
IGSF1	Hemizygote type	Other related disease	XLR	Maternal heterozygosis
KCNQ5	Heterozygosis type	OMIM: 617601	AD	Paternal heterozygosis
KIF1A	Heterozygosis type	OMIM: 614255	AD	Paternal heterozygosis
KMT2C	Deletion type	OMIM: 617768	AD	De novo variation
KMT2D	Heterozygosis type	OMIM: 147920	AD	Maternal heterozygosis
LAGE3	Hemizygote type	OMIM: 301006	XLR	Maternal heterozygosis
LBR	Heterozygosis type	OMIM: 169400	AD	Maternal heterozygosis
LHX3	Isozygoty type	OMIM: 221750	AR	Parental hybridization
LONP1	Heterozygosis type	OMIM: 600373	AR	Uninspected source
LTBP3	Heterozygosis type	OMIM: 617809	AD	Uninspected source
MAP2K2	Heterozygosis type	OMIM: 615280	AD	Maternal heterozygosis
MATN3	Heterozygosis type	OMIM: 607078	AD	Paternal heterozygosis
MBTPS2	Hemizygote type	OMIM: 301014	XLR	Maternal heterozygosis
MRAS	Heterozygosis type	OMIM: 618499	AD	Paternal heterozygosis
MYO5A	Heterozygosis type	OMIM: 214450	AR	Paternal heterozygosis
NF1	Heterozygosis type	OMIM: 162210	AD	De novo variation
NFE2L2	Heterozygosis type	OMIM: 617744	AD	Maternal heterozygosis
NIPBL	Heterozygosis type	OMIM: 122470	AD	Maternal heterozygosis
NPR2	Heterozygosis type	OMIM: 616255	AD	Paternal heterozygosis
OBSL1	Isozygoty type	Other related disease	AR	Parental hybridization
PDE4D	Heterozygosis type	OMIM: 614613	AD	Paternal heterozygosis
PHEX	Hemizygote type	OMIM: 307800	XLD	Maternal heterozygosis
PIEZO2	Heterozygosis type	Not reported	Not reported	Maternal heterozygosis
PNPLA2	Heterozygosis type	Not reported	Not reported	Maternal heterozygosis
POLA1	Hemizygote type	OMIM: 301030	XLR	Maternal heterozygosis
POLR1A	Heterozygosis type	OMIM: 616462	AD	Maternal heterozygosis
PQBP1	Hemizygote type	OMIM: 309500	XLR	Maternal heterozygosis
PRMT7	Heterozygosis type	Other related disease	AR	Uninspected source
PTH1R	Heterozygosis type	OMIM: 156400	AD	Paternal heterozygosis
RAF1	Heterozygosis type	OMIM: 611553	AD	Non-paternal heterozygosis
RECQL4	Heterozygosis type	Other related disease	AR	Maternal heterozygosis
SAMD9	Heterozygosis type	OMIM: 617053	AD	Maternal heterozygosis
SETD2	Heterozygosis type	OMIM: 616831	AD	Paternal heterozygosis
SHH	Heterozygosis type	Other related disease	AD	Paternal heterozygosis
SHOX	Heterozygosis type	OMIM: 249700	PR	Maternal heterozygosis
SLC6A8	Hemizygote type	OMIM: 300352	XLR	Maternal heterozygosis
SMARCA2	Heterozygosis type	OMIM: 601358	AD	Paternal heterozygosis
SMS	Hemizygote type	Other related disease	XLR	Maternal heterozygosis
SON	Heterozygosis type	OMIM: 617140	AD	Paternal heterozygosis
SOX11	Heterozygosis type	OMIM: 615866	AD	Maternal heterozygosis
SOX4	Heterozygosis type	Other related disease	AD	De novo variation
SP7	Isozygoty type	OMIM: 613849	AR	Parental hybridization
STAT5B	Heterozygosis type	OMIM: 245590	AD/AR	Paternal heterozygosis
TBX1	Heterozygosis type	OMIM: 188400	AD	Non-paternal heterozygosis
TRPS1	Heterozygosis type	OMIM: 190350	AD	Paternal heterozygosis
TRPV4	Heterozygosis type	OMIM: 113500	AD	Paternal heterozygosis
WWOX	Heterozygosis type	Other related disease	AR	Maternal heterozygosis
ZC4H2	Deletion type	OMIM: 314580	XLR	Maternal heterozygosis

### 3.2 Possible pathogenic gene mutation

**Figure 1:**
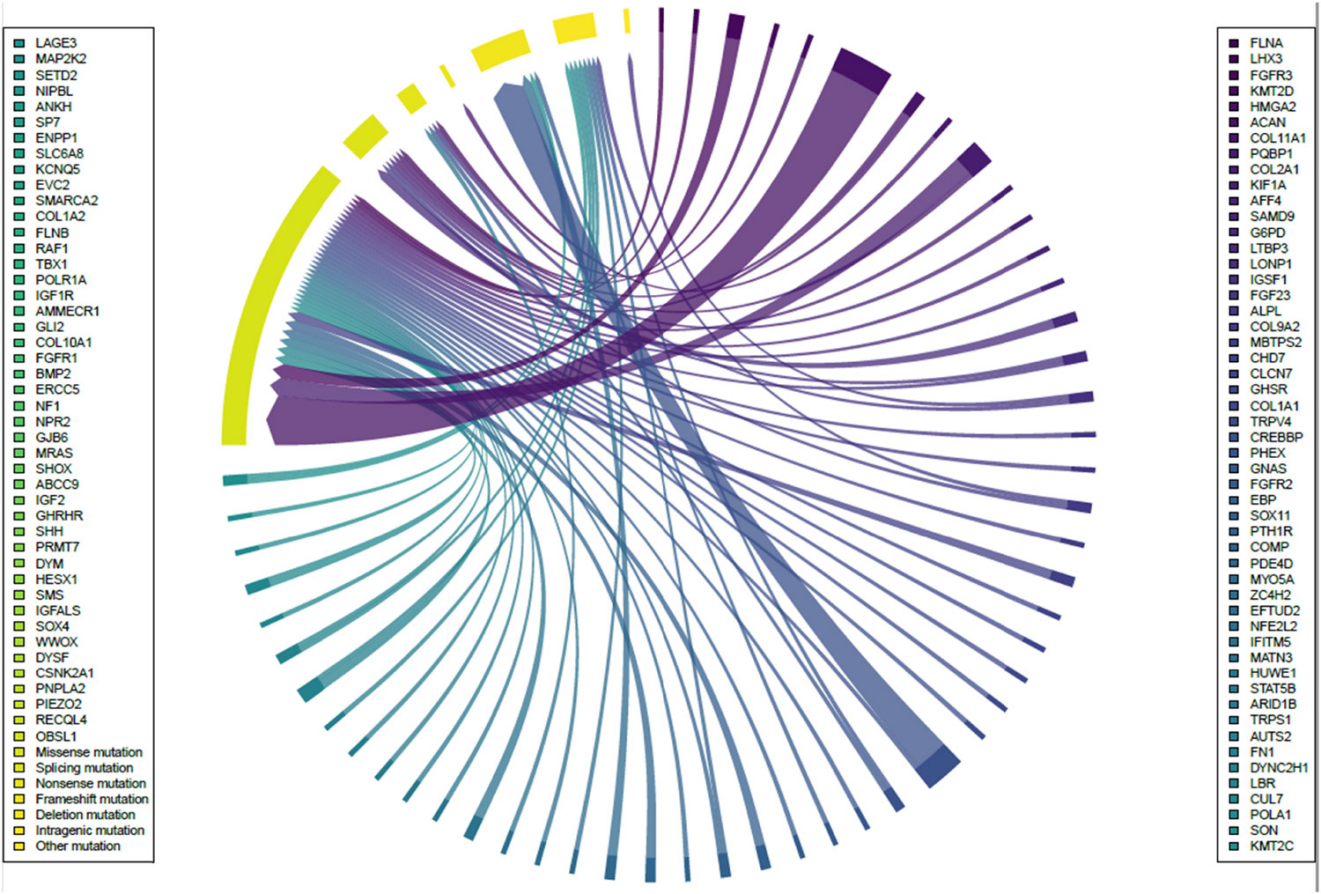
Possible pathogenic gene mutation and its variation classification.

A total of 97 different gene mutations were discovered among the 56 subjects included in the study. The top three most frequent gene mutations were ACAN, PHEX, and COL2A1. Of these potential pathogenic genes, missense mutation was the most common mutation type, followed by deletion mutation, intragenic mutation, splicing mutation, nonsense mutation, frameshift mutation, and other mutations (Figure 1).

### 3.3 Copy number variations (CNV) of possible pathogenic genes

**Figure 2:**
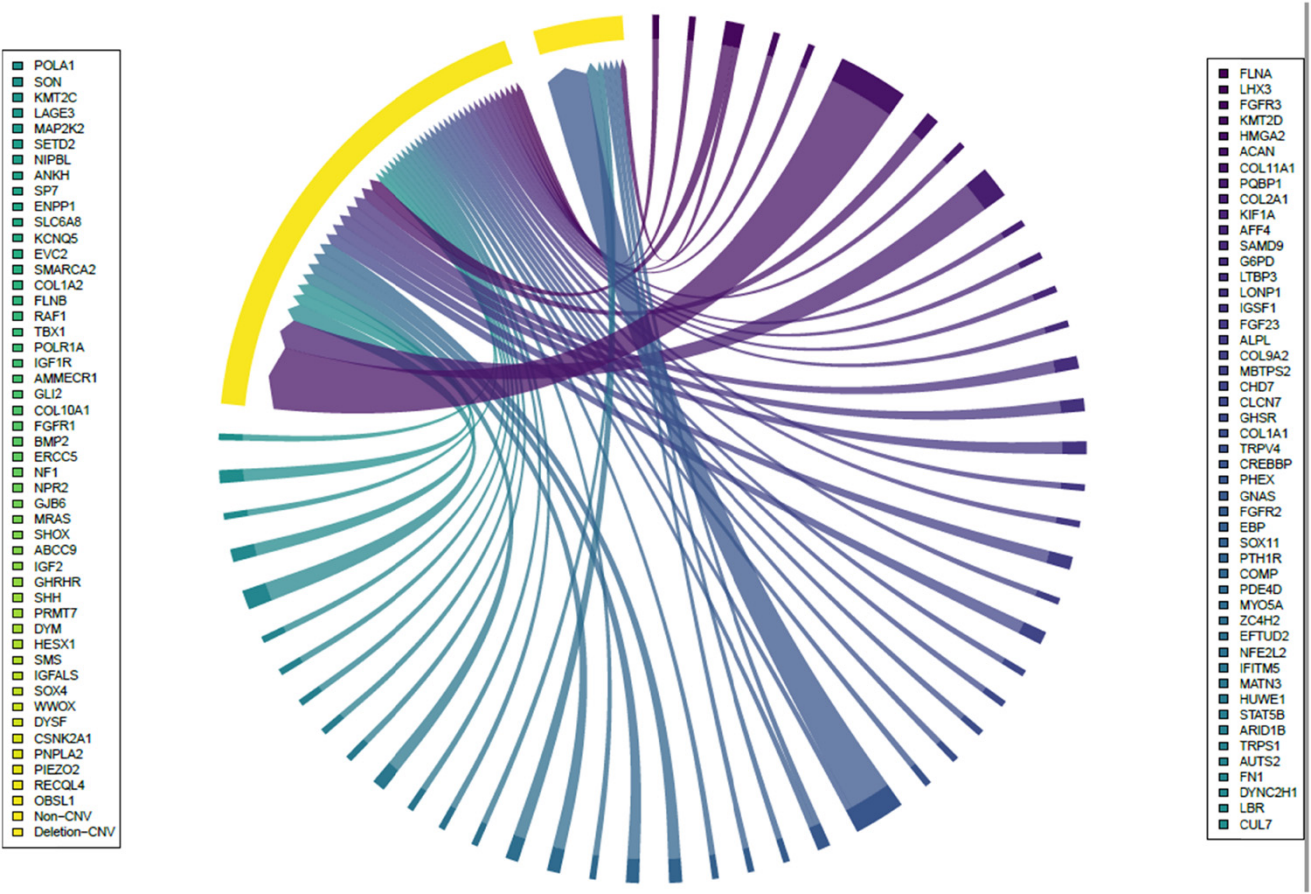
Copy number variations of possible pathogenic genes.

CNV is a type of genetic variation that involves alterations in the number of copies of a specific segment of DNA. Normally, a particular DNA segment exists in two copies to correspond with the common diploid human body. However, in the case of genotype copy number variation, this number could range from one to three or even higher. CNV can vary in size, ranging from 1 kilobase (kb) to several megabases (Mb). Among these potential pathogenic genes, both non-CNV and deletion-CNV were identified. It is worth noting that deletion-CNV was most closely associated with PHEX (Figure 2 and Table 1).

### 3.4 Prediction of protein functional damage caused by these possible pathogenic gene mutations

**Figure 3:**
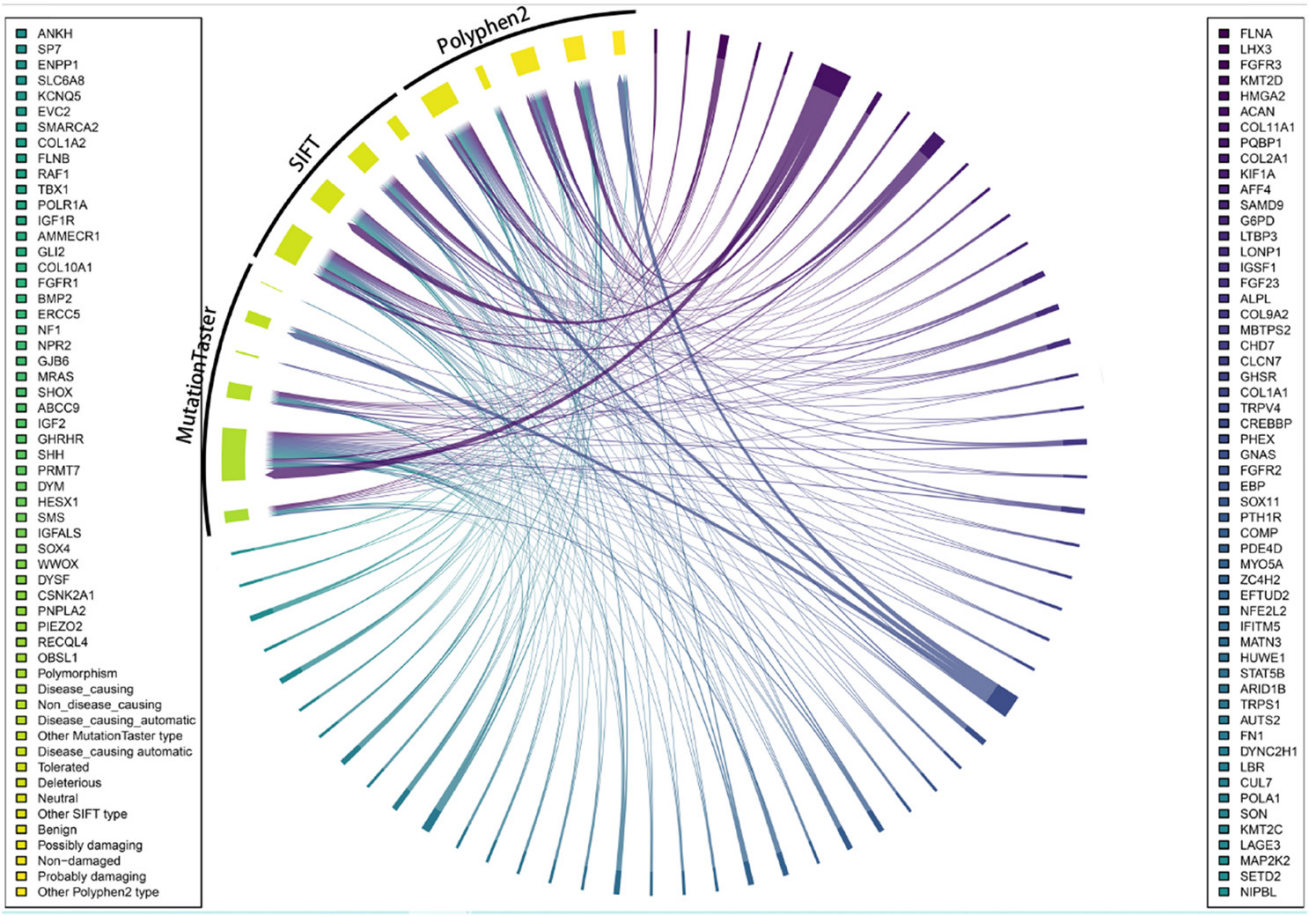
Prediction of protein functional damage caused by these possible pathogenic gene mutations.

To assess the protein functional impact of these potential pathogenic gene mutations, analyses were performed utilizing MutationTaster, SIFT, and PolyPhen-2. The results of the MutationTaster analysis revealed a substantial increase in the disease-causing protein functional damage. On the other hand, SIFT analysis revealed a predominant enrichment of tolerated protein functional damage. In addition, PolyPhen-2 analysis revealed a significant prevalence of functional damage to benign proteins (Figure 3).

### 3.5 The chromosomal location of possible pathogenic genes

**Figure 4:**
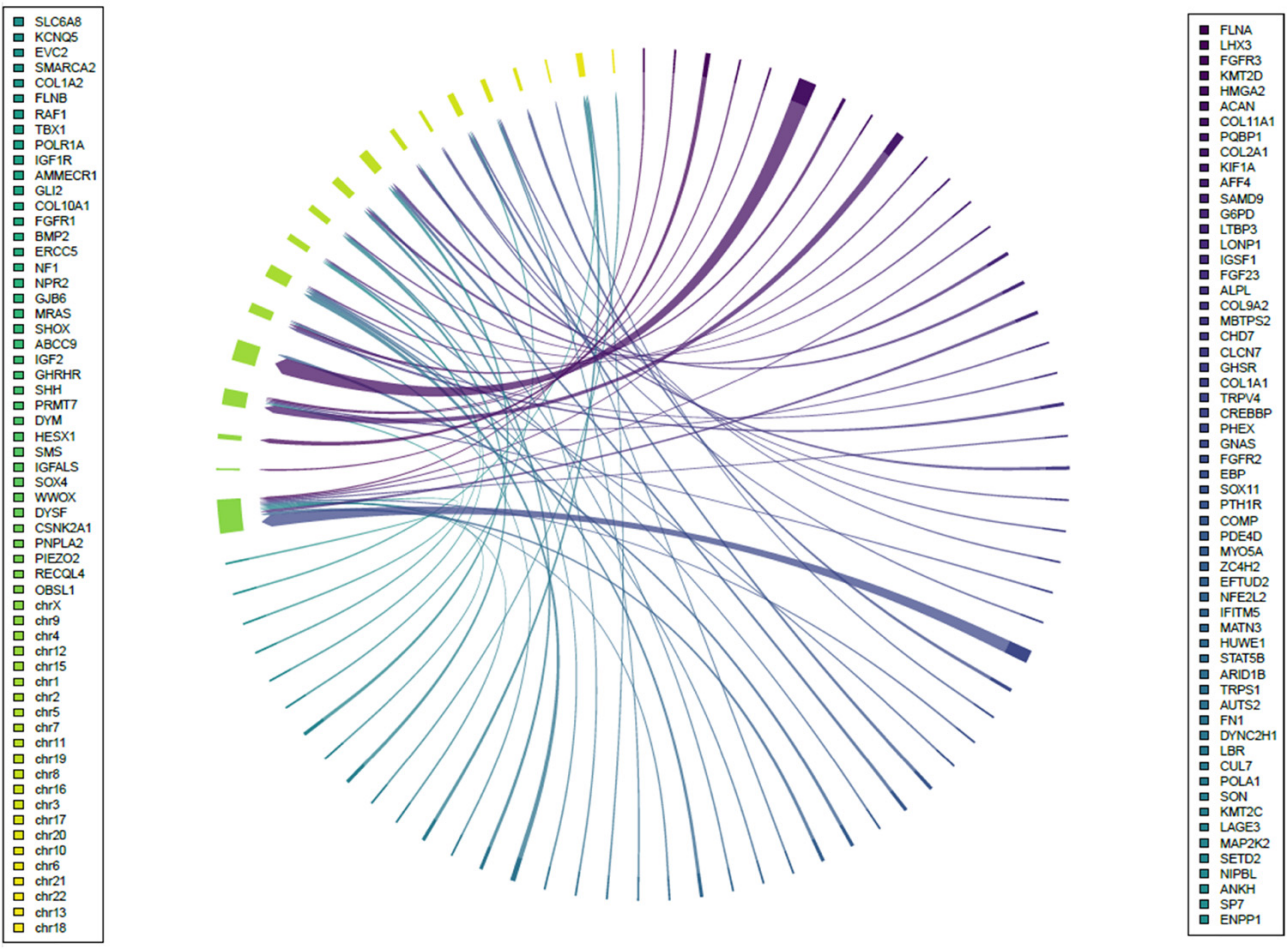
The chromosomal location of possible pathogenic genes.

Figure 4 displays the chromosomal locations that exhibited the highest enrichment of potential pathogenic genes, with chr12, chr5, and chr2 being the top three. Furthermore, Table 2 provides detailed information on the nucleotide alterations and corresponding amino acid changes of these potential pathogenic genes.

**Table 2:** The nucleotide alteration and amino acid change of possible pathogenic genes

Gene	Transcript number	Chromosomal location	Nucleotide alteration	Amino acid change
ABCC9	NM_005691	chr12	c.2005G>A	p.Asp669Asn
ACAN	NM_013227	chr15	c.571G>A	p.Ala191Thr
			c.1546G>A	p.Ala516Thr
			c.7246G>T	p.Gly2416Cys
			c.6530T>C	p.Val2177Ala
			c.1546G>A	p.Ala516Thr
			c.7039G>A	p.Glu2347Lys
			c.7267G>A	p.Glu2423Lys
AFF4	NM_014423	chr5	c.2934-6C>T	No amino acid change
ALPL	NM_000478	chr1	c.398C>G	p.Ala133Gly
AMMECR1	NM_015365	chrX	c.888-15C>T	No amino acid change
ANKH	NM_054027	chr5	c.688-14G>A	No amino acid change
ARID1B	NM_020732	chr6	c.2358G>A	p.Met786Ile
AUTS2	NM_015570	chr7	c.2531 + 4C>T	No amino acid change
BMP2	NM_001200	chr20	c.482T>C	p.Leu161Ser
CHD7	NM_017780	chr8	c.6571G>A	p.Glu2191Lys
			c.7471C>T	p.Arg2491Cys
CLCN7	NM_001287	chr16	c.1226G>A	p.Arg409Gln
COL10A1	NM_000493	chr6	c.382G>A	p.Asp128Asn
COL11A1	NM_080629	chr1	c.2079 + 8G>A	No amino acid change
			c.475A>G	p.Ile159Val
COL1A1	NM_000088	chr17	c.3310G>A	p.Asp1104Asn
COL1A2	NM_000089	chr7	c.2482G>T	p.Val828Phe
			c.892-13C>G	No amino acid change
COL2A1	NM_001844	chr12	c.3327 + 3G>A	No amino acid change
			c.3107G>A	p.Arg1036Gln
			c.1913C>T	p.Thr638Ile
			c.17C>A	p.Ala6Asp
			c.580G>A	p.Ala194Thr
COL9A2	NM_001852	chr1	c.847-9G>A	No amino acid change
			c.847-8C>T	No amino acid change
COMP	NM_000095	chr19	c.344C>G	p.Pro115Arg
			c.218-7C>G	No amino acid change
CREBBP	NM_004380	chr16	c.6479C>T	p.Ala2160Val
CSNK2A1	NM_001895.3	chr20	c.832C>T	p.Arg278*
CUL7	NM_014780	chr6	c.4996G>A	p.Gly1666Ser
			c.3274C>T	p.Arg1092Trp
DYM	NM_017653.5	chr18	c.113C>T	p.Ser38Leu
DYNC2H1	NM_001080463	chr11	c.8833-3T>C	No amino acid change
			c.5177G>A	p.Arg1726Gln
DYSF	NM_003494.4	chr2	c.937 + 1G>A	Amino acid deficiency
EBP	Non-transcript	chrX	Nucleotide deletion	Amino acid deficiency
EFTUD2	NM_004247	chr17	c.1962 + 4C>G	No amino acid change
ENPP1	NM_006208	chr6	c.2335A>C	p.Thr779Pro
ERCC5	NM_000123	chr13	c.760A>G	p.Met254Val
			c.1031C>T	p.Thr344Ile
EVC2	NM_147127	chr4	c.1456C>T	p.Arg486Cys
FGF23	NM_020638	chr12	c.559C>G	p.Arg187Gly
FGFR1	NM_001354369	chr8	c.1273-15C>T	No amino acid change
FGFR2	NM_022970	chr10	c.1352G>A	p.Arg451His
FGFR3	NM_001163213	chr4	c.1319T>C	p.Val440Ala
			c.2207C>T	p.Ala736Val
			c.65C>T	p.Ser22Leu
FLNA	NM_001110556	chrX	c.2522G>A	p.Arg841Gln
FLNB	NM_001457	chr3	c.6680C>G	p.Ser2227Cys
			c.1409G>T	p.Arg470Leu
FN1	NM_212482	chr2	c.3307A>C	p.Ile1103Leu
			c.1547-6A>G	No amino acid change
			c.3307A>C	p.Ile1103Leu
G6PD	NM_000402	chrX	c.1466G>T	p.Arg489Leu
GHRHR	NM_000823.4	chr7	c.269-16C>T	Amino acid deficiency
GHSR	NM_198407	chr3	c.829C>T	p.Leu277Phe
GJB6	NM_006783	chr13	c.228del	p.Trp77Glyfs*5
GLI2	NM_005270.4	chr2	c.3677G>A	p.Arg1226Gln
			c.4627C>T	p.Arg1543Cys
GNAS	NM_080425	chr20	c.730C>T	p.Arg244Ter
			c.2361 + 1G>A	No amino acid change
HESX1	NM_003865.3	chr3	c.68T>C	p.lle23Thr
HMGA2	NM_001300919	chr12	c.249G>A	p.Trp83Ter
HUWE1	NM_031407	chrX	c.5150A>G	p.Asn1717Ser
IFITM5	NM_001025295	chr11	c.190C>T	p.Arg64Ter
IGF1R	NM_000875	chr15	c.4012G>A	p.Ala1338Thr
IGF2	NM_000612.6	chr11	c.100G>A	p.Gly34Ser
IGFALS	NM_004970.2	chr16	c.1794C>A	p.Ser598Arg
			c.418G>A	p.Gly140Ser
IGSF1	NM_001170961.1	chrX	c.2623 + 1G>C	Amino acid deficiency
			c.1030C>T	p.Arg344Ter
KCNQ5	NM_001160133	chr6	c.2167G>A	p.Ala723Thr
KIF1A	NM_001244008	chr2	c.3680C>T	p.Pro1227Leu
KMT2C	Non-transcript	chr7	Nucleotide deletion	Amino acid deficiency
			c.11961-14T>G	No amino acid change
KMT2D	NM_003482	chr12	c.5645-3C>T	No amino acid change
LAGE3	NM_006014	chrX	c.139A>G	p.Arg47Gly
LBR	NM_002296	chr1	c.1757G>A	p.Arg586His
LHX3	NM_014564	chr9	c.979G>A	p.Ala327Thr
LONP1	NM_004793	chr19	c.2155-7C>T	No amino acid change
			c.2392G>A	p.Gly798Ser
LTBP3	NM_001130144	chr11	c.2977 + 10C>G	No amino acid change
			c.2977 + 10C>G	No amino acid change
MAP2K2	NM_030662	chr19	c.238G>A	p.Ala80Thr
			c.893C>T	p.Pro298Leu
MATN3	NM_002381	chr2	c.838A>C	p.Ile280Leu
			c.209G>A	p.Arg70His
MBTPS2	NM_015884	chrX	c.1060C>T	p.Arg354Cys
MRAS	NM_012219	chr3	c.226G>A	p.Ala76Thr
MYO5A	NM_000259	chr15	c.1841G>A	p.Arg614Gln
			c.2477G>A	p.Arg826His
NF1	NM_000267	chr17	c.2294G>A	p.Arg765His
			c.4890dup	p.Asn1631Glnfs*4
NFE2L2	NM_006164	chr2	c.160C>T	p.Leu54Phe
NIPBL	NM_133433	chr5	c.3068A>G	p.Lys1023Arg
NPR2	NM_003995	chr9	c.328C>T	p.Arg110Cys
OBSL1	NM_015311.2	chr2	c.458dupG	p.Leu154fs
PDE4D	NM_001197218	chr5	c.59G>A	p.Cys20Tyr
PHEX	NM_000444	chrX	Nucleotide deletion	Amino acid deficiency
			c.1619T>C	p.Phe540Ser
PIEZO2	NM_022068.3	chr18	c.668G>A	p.Gly223Glu
PNPLA2	NM_020376.3	chr11	c.1090C>T	p.Arg364Trp
			c.1466G>T	p.Ser489Ile
POLA1	NM_016937	chrX	c.3844C>T	p.Pro1282Ser
POLR1A	NM_015425	chr2	c.1866 + 3A>G	No amino acid change
PQBP1	NM_001032383	chrX	c.451_454del	p.Arg153SerfsTer41
PRMT7	NM_019023.4	chr16	c.283-16C>T	Amino acid deficiency
PTH1R	NM_000316	chr3	c.182G>A	p.Ser61Asn
RAF1	NM_002880	chr3	c.778A>C	p.Thr260Pro
RECQL4	NM_004260.3	chr8	c.3133G>A	p.Ala1045Thr
			c.901G>A	p.Glu301Lys
SAMD9	NM_001193307	chr7	c.2564A>G	p.Gln855Arg
SETD2	NM_014159	chr3	c.6746A>G	p.His2249Arg
SHH	NM_000193.4	chr7	c.1189C>T	p.Arg397Cys
SHOX	NM_000451	chrX	c.278-15C>A	No amino acid change
SLC6A8	NM_005629	chrX	c.1021G>A	p.Ala341Thr
SMARCA2	NM_003070	chr9	c.4589C>G	p.Ser1530Cys
SMS	NM_004595.5	chrX	c.211>G	p.Leu71Val
SON	NM_032195	chr21	c.2395A>G	p.Ser799Gly
SOX11	NM_003108	chr2	c.674A>G	p.Asp225Gly
SOX4	NM_003107.3	chr6	c.192C>G	p.Asn64Lys
SP7	NM_001173467	chr12	c.565C>T	p.Pro189Ser
STAT5B	NM_012448	chr17	c.1696C>T	p.Arg566Trp
TBX1	NM_080647	chr22	c.463G>A	p.Asp155Asn
TRPS1	NM_014112	chr8	c.1244C>T	p.Thr415Ile
TRPV4	NM_021625	chr12	c.760G>C	p.Val254Leu
WWOX	NM_016373.4	chr16	c.411G>T	p.Gly137Val
			c.1078G>A	p.Val360Met
ZC4H2	Non-transcript	chrX	Nucleotide deletion	Amino acid deficiency

* indicate statistical significance (*P < 0.05).

## 4. Discussion

The comprehensive genomic survey presented in this study identified a diverse range of genetic variations in pediatric patients with short stature. The study revealed a total of 97 gene mutations, with the most common mutations found in genes like ACAN, PHEX, and COL2A1. These variations included missense, deletion, splicing, and nonsense mutations, along with copy number variations, potentially impacting protein function and physiological processes. These genetic alterations have the potential to significantly influence biological systems and human health, underscoring the importance of understanding the genetic basis of short stature for clinical management and therapeutic strategies. The rationale for the study stems from the significant impact of genetic variations on human health, particularly in the context of conditions such as short stature, diabetes, and cancer. It is vital to comprehend the particular genetic variations selected for examination and their significance to human health to clarify their possible consequences and develop personalized healthcare strategies. Due to their capacity to affect vital cellular processes and contribute to susceptibilities to disease, the genetic variations selected for investigation, including missense mutations, nonsense mutations, and silent mutations are of the utmost importance to human health. These variations were selected based on their known associations with regulatory pathways governing skeletal growth, insulin signaling, and cellular proliferation, all of which have direct implications for physiological well-being and disease states. Genetic variations and short stature: Short stature is a prevalent issue in the field of pediatric medicine, with a significant number of cases thought to be caused by genetic factors. The genes that control the growth and development of the skeleton, specifically those involved in the GHRH-GH-IGF-1 pathway, have a significant impact on an individual’s stature. Mutations in these genes, including missense mutations, can directly impact skeletal growth and developmental pathways, making them pertinent targets for investigation in the context of short stature.

Numerous missense mutations were identified in genes including HUWE1 (NM_031407), IGF1R (NM_000875), and IGF2 (NM_000612.6), among others. Missense mutations result in the change of a single nucleotide, leading to a different amino acid in the protein sequence. The resultant proteins may experience substantial structural and functional changes due to these substitutions, which may affect the biological processes that they regulate. For example, HUWE1 (HECT, UBA, and WWE domain containing 1) is a gene known to be involved in protein ubiquitination, a process critical for regulating diverse cellular functions. Mutations in this gene have been associated with X-linked mental retardation and may contribute to oncogenesis in various tissues, but there is no direct correlation provided between HUWE1 missense mutations and short stature. Mutations in the IGF1R gene, which encodes the insulin-like growth factor 1 receptor, could potentially disrupt insulin signaling and glucose metabolism, contributing to diseases like diabetes or cancer. Some of the identified missense mutations could affect genes involved in insulin signaling pathways, such as the IGF1R [25]. Missense mutations can cause a functional disruption of IGF1R, which can result in altered insulin signaling. This can have an impact on glucose metabolism and insulin sensitivity. These alterations may potentially contribute to the onset of insulin resistance, which is a defining characteristic of type 2 diabetes. Besides, missense mutations in genes related to glucose metabolism, such as IGF2, may perturb normal glucose homeostasis [26]. Similarly, mutations in IGF2, an important growth factor in human development and growth [27,28], may cause abnormal growth and development, and potentially contribute to the onset of diseases such as Beckwith-Wiedemann syndrome or cancer. sVariations in the function of IGF2 may have an impact on the utilization and regulation of glucose, which may have implications for the risk of developing diabetes. Missense mutations in genes involved in cell signaling pathways, such as IGF1R, can impact cellular processes related to proliferation and survival. Dysregulated IGF1R signaling due to missense mutations can contribute to uncontrolled cell growth and reduced apoptosis, which are characteristic features of cancer development and progression.

Most notably, the ZNF764 (XM_001300919) and IFITM5 (NM_001025295) genes have been identified to contain nonsense mutations. The introduction of premature stop codons into the coding sequence by these mutations almost certainly results in a truncated protein product. Depending on the location of the truncation, the protein may completely lose its functional capability or gain novel, inappropriate functions, a phenomenon known as gain-of-function mutation [29,30]. Some of these mutations have the potential to cause severe damage, manifesting as disease states or aberrant cellular behavior. When examining the nonsense mutations that have been identified in ZNF764 and IFITM5, it is critical to consider the possible mechanisms by which truncated protein products could acquire inappropriate and novel functions. Additionally, it is crucial to consider the implications of these mechanisms on cellular behavior and disease states. Novel functions of truncated proteins may manifest as aberrant interactions, altered subcellular localization, or dominant-negative interference. Additionally, these mutations can lead to dysregulated signaling pathways, disruption of protein complexes, and altered gene expression.

Notably, silent mutations have been detected in our dataset as well, specifically in the KMT2C and POLR1A genes. Silent mutations, also known as synonymous mutations, are DNA sequence changes that do not result in an alteration of the encoded amino acid within the corresponding protein [31]. Traditionally, silent mutations were regarded as benign conditions that did not affect the function of proteins. On the other hand, recent studies have revealed that these mutations have the potential to impact protein synthesis and overall cellular function by causing disruptions in splicing regulation, gene expression, and mRNA stability [32]. Silent mutations can influence gene expression levels by altering codon usage, affecting the rate of protein translation, and potentially impacting overall protein abundance. Furthermore, these mutations have the potential to affect the structure and stability of mRNA, which may affect the efficacy of mRNA degradation and processing. Furthermore, it has been discovered that it exerts an impact on alternative splicing, thereby influencing the synthesis of distinct protein isoforms from a single gene. An investigation into the inherited blood disorder beta-thalassemia demonstrates how silent mutations can affect splicing efficacy, ultimately resulting in the onset of the disease [33]. These silent mutations affect the splicing of pre-mRNA, resulting in aberrant splicing patterns and the production of abnormal hemoglobin, contributing to the pathogenesis of beta-thalassemia. Further research is needed to determine the exact effects of silent mutations in the POLR1A and KMT2C genes, however, when evaluating their significance to disease processes, it is crucial to take into account the possible regulatory functions of these mutations in gene expression and protein synthesis.

The present investigation identified particularly noteworthy mutations in the genes PHEX (NM_000444), ZC4H2 (chrX), and KMT2C. These mutations led to a condition known as ‘amino acid deficiency’. Mutations of PHEX and ZC4H2, it likely indicate missing segments in the amino acid sequence due to nucleotide deletions [34]. Amino acid deficiencies arising from nucleotide deletions can have a direct impact on the functionality of the encoded proteins in the PHEX and ZC4H2 contexts, which are associated with X-linked hypophosphatemia and Wieacker-Wolff syndrome, respectively. This may disrupt critical biological pathways, leading to the characteristic signs and symptoms associated with these genetic disorders. Further investigation is required to definitively identify what is meant by ‘amino acid deficiency’ and the implication it has on protein function and overall physiology.

Furthermore, synonymous mutations or mutations resulting in an apparent “no amino acid change” were observed, suggesting that these genetic changes do not cause a modification to the sequence of the encoded protein. It has been widely accepted that these mutations remained “silent” and had no discernible impact [35]. However, we now know, that synonymous mutations can influence gene expression levels, alter mRNA stability and structure, or affect splicing regulation. Hence, these ‘innocuous’ mutations might have been overlooked and require thorough investigation.

In earlier research, Chen and colleagues [10] identified 24 potentially harmful or detrimental variants of collagen genes in patients exhibiting skeletal abnormalities and short stature; COL2A1 mutations were the most prevalent, accounting for around 57.7% of each case. Additionally, they identified prevalent mutations associated with skeletal development, encapsulating FGFR3, COMP, NPR2, ACAN, and FBN1. These results have a few similarities to this study, however, this study further added to their study by finding a series of new possible pathogenic gene mutations and presenting the gene feature of Chinese patients with short stature.

The identified genetic alterations discovered in this study represent novel and significant findings with potential implications for both diagnostic and therapeutic applications in a clinical setting. The diversity of genetic variations, including missense, deletion, splicing, and nonsense mutations, presents a wealth of potential targets for further exploration in the context of personalized medicine for individuals with short stature. Particularly in individuals with growth-related disorders, the existence of missense mutations in genes including IGF1R, IGF2, and HUWE1 holds potential for the advancement of targeted therapies. The identified mutations may have clinical significance across a range of growth-related disorders, as they have been linked to growth failure, developmental disorders, and X-linked mental retardation. Additionally, the presence of silent mutations and their impact on gene expression, mRNA stability, and splicing regulation suggests the potential for personalized treatment approaches tailored to the specific genetic profile of individuals with short stature. Utilizing the identified genetic alterations as diagnostic markers could enable more precise and individualized diagnostics, leading to earlier detection and intervention for individuals with underlying genetic causes of short stature. Furthermore, the potential therapeutic relevance of the identified genetic alterations extends to the development of novel treatment modalities, including gene therapy and targeted pharmacological interventions. By understanding the specific genetic variations contributing to short stature in individual patients, tailored therapeutic strategies could be developed to address specific molecular defects, potentially leading to more effective treatments and improved clinical outcomes. The implications of these findings extend beyond short stature, as the genetic variations identified in this study may also have relevance to a broader range of growth and developmental disorders. The discovery could greatly benefit our understanding of short stature and the development of personalized treatment methods for many different genetic disorders that impair growth and development by revealing the genetic basis of these conditions. Therefore, additional studies are needed to confirm the functional effects of the found genetic variations, particularly in the context of short stature and related growth and developmental disorders. Experimental validation is crucial to elucidate the specific consequences of the identified mutations on protein function, cellular processes, and overall physiological outcomes.

Despite the comprehensive nature of the genomic survey conducted in this study, several inherent limitations need to be addressed. First, a significant constraint is the absence of functional validation for the identified mutations. Although various genetic variations, such as missense, deletion, splicing, and nonsense mutations, were effectively identified in the study, their experimental validation did not establish their functional impact on protein activity or cellular function. This limitation hinders the ability to definitively assess how these mutations may alter biological processes and contribute to disease susceptibility or pathogenesis. Additionally, the study did not consider the interplay with the epigenetic landscape. Epigenetic factors, such as DNA methylation and histone modifications, can modulate gene expression independently of changes in the DNA sequence. The potential regulatory effects on protein function and gene expression were not taken into consideration due to the lack of analysis concerning epigenetic modifications. Considering the significant role of epigenetics in regulating gene expression and cellular behavior, this oversight limits the comprehensive understanding of the genetic contributions to short stature. Furthermore, the study did not address the potential interactions between multiple mutations within the same individual. It is increasingly recognized that the cumulative effects of multiple mutations may have synergistic or antagonistic impacts on protein function and cellular pathways. Understanding the potential interactions between different mutations is vital for unraveling the complex genetic architecture underlying short stature. Therefore, the lack of consideration for potential epistatic interactions between mutations represents a notable limitation in the interpretation of the results. These limitations collectively impact the interpretation of the results by highlighting the need for caution when drawing direct associations between the identified mutations and the observed phenotypes. Without functional validation, insights into the specific consequences of the mutations on protein function and cellular processes remain speculative. Additionally, the absence of considerations regarding the epigenetic landscape and potential interactions between multiple mutations may result in an incomplete representation of the genetic contributions to short stature, potentially leading to oversimplified or erroneous conclusions regarding the genetic etiology of the condition.

## 5. Conclusion

In summary, our investigation reveals an extensive range of genetic variations that could have various and significant implications for short stature. The results of this study pave the way for future research to explore the functional implications of these genetic variations and evaluate their potential as therapeutic targets or diagnostic indicators. However, the incorporation of these genetic alterations into predictive or therapeutic models necessitates careful consideration, considering the possible modification of their effects by other genetic or epigenetic factors, environmental influences, or biological randomness, which still requires further comprehension.

## References

[1] Allen DB. Addressing short stature is still a tall order. J Pediatr. 2023;262:113659. 10.1016/j.jpeds.2023.113659 37543284

[2] Butler MG, Miller BS, Romano A, Ross J, Abuzzahab MJ, Backeljauw P, et al. Genetic conditions of short stature: A review of three classic examples. Front Endocrinol (Lausanne). 2022;13:1011960. 10.3389/fendo.2022.1011960 PMC9634554 36339399

[3] Karaoglan M. Short stature due to bioinactive growth hormone (kowarski syndrome). Endocr Pract. 2023;29:902–11. 10.1016/j.eprac.2023.08.013 37657628

[4] Mastromauro C, Giannini C, Chiarelli F. Short stature related to growth hormone insensitivity (GHI) in childhood. Front Endocrinol (Lausanne). 2023;14:1141039. 10.3389/fendo.2023.1141039 PMC10050683 37008935

[5] McDonald EJ, De Jesus O. Achondroplasia. Treasure Island (FL), StatPearls Publishing. 2022. 32644689

[6] Panigrahi I, Kaur P, Chaudhry C, Shariq M, Naorem DD, Gowtham BC, et al. Short stature syndromes: case series from India. J Pediatr Genet. 2022;11(4):279–86. 10.1055/s-0041-1726037 PMC9578783 36267864

[7] Rajkumar V, Waseem M. Familial short stature. Treasure Island (FL): StatPearls Publishing LLC; 2020. 32644549

[8] Rani D, Shrestha R, Kanchan T, Krishan K. Short stature. Treasure Island (FL): StatPearls Publishing; 2020. 32310491

[9] Shemesh A, Margolin E. Kearns-Sayre syndrome. Treasure Island(FL): StatPearls Publishing; 2018. 29493966

[10] Chen M, Miao H, Liang H, Ke X, Yang H, Gong F, et al. Clinical characteristics of short-stature patients with collagen gene mutation and the therapeutic response to rhGH. Front Endocrinol (Lausanne). 2022;13:820001. 10.3389/fendo.2022.820001 PMC8889571 35250876

[11] Zadik Z, Zelinska N, Iotova V, Skorodok Y, Malievsky O, Mauras N, et al. An open-label extension of a phase 2 dose-finding study of once-weekly somatrogon vs once-daily Genotropin in children with short stature due to growth hormone deficiency: results following 5 years of treatment. J Pediatr Endocrinol Metab. 2023;36(3):261–9. 10.1515/jpem-2022-0359 36732285

[12] Fermin Gutierrez MA, Mendez MD. Prader-Willi Syndrome. Treasure Island (FL): StatPearls Publishing; 2020. 31985954

[13] Lin AE, Brunetti-Pierri N, Lindsay ME, Schimmenti LA, Starr LJ. Myhre syndrome. Seattle, WA: University of Washington; 2017. 28406602

[14] Luo X, Zhao S, Yang Y, Dong G, Chen L, Li P, et al. Long-acting PEGylated growth hormone in children with idiopathic short stature. Eur J Endocrinol. 2022;187(5):709–18. 10.1530/EJE-22-0449 36130048

[15] Maghnie M, Ranke MB, Geffner ME, Vlachopapadopoulou E, IbÃ¡Ã ±EZL, Carlsson, M, et al. Safety and efficacy of pediatric growth hormone therapy: results from the full KIGS cohort. J Clin Endocrinol Metab. 2022;107(12):3287–301. 10.1210/clinem/dgac517 PMC9693805 36102184

[16] Mauras N, Ross J, Mericq V. Management of growth disorders in puberty: GH, GnRHa, and aromatase inhibitors: A clinical review. Endocr Rev. 2023;44(1):1–13. 10.1210/endrev/bnac014 35639981

[17] Öztürk AP, Aslanger AD, Bas F, Toksoy G, Karaman V, Bagirova G, et al. Phenotype-genotype correlations of GH1 gene variants in patients with isolated growth hormone deficiency (IGHD) or multiple pituitary hormone deficiency (MPHD). Horm Res Paediatr. 2024;97:126–33. 10.1159/000531113 PMC11126197 37315542

[18] Eren E, Ongen YD, Ozgur T, Ozpar R, Demirbas O, Yazici Z, et al. Normal or elevated prolactin is a good indicator to show pituitary stalk interruption syndrome in patients with multiple pituitary hormone deficiency. J Pediatr Endocrinol Metab. 2022;35(11):1394–400. 10.1515/jpem-2022-0366 36136319

[19] Huang Q, Xu H, Wang X, Mao J, Yu B, Zhu Y, et al. Relationship between growth hormone deficiency and nonalcoholic fatty liver disease in patients with pituitary stalk interruption syndrome. Clin Endocrinol (Oxf). 2022;97(5):612–21. 10.1111/cen.14732 35384023

[20] Jakobsen LK, Jensen RB, Birkebæk NH, Hansen D, Christensen AR, Bjerrum MC, et al. Diagnosis and incidence of congenital combined pituitary hormone deficiency in Denmark – a national observational study. J Clin Endocrinol Metab. 2023;1008:2475–85. 10.1210/clinem/dgad198 PMC10505542 37043518

[21] Kardelen AD, Najafli A, BaÅŸ F, Karaman B, Toksoy G, PoyrazoÄŸlu Å, et al. PROKR2 mutations in patients with short stature who have isolated growth hormone deficiency and multiple pituitary hormone deficiency. J Clin Res Pediatr Endocrinol. 2023;15:338–47. 10.4274/jcrpe.galenos.2023.2023-4-4 PMC10683534 37338295

[22] Stagi S, Tufano M, Chiti N, Cerutti M, Li Pomi A, Aversa T, et al. Management of neonatal isolated and combined growth hormone deficiency: current status. Int J Mol Sci. 2023;24(12):10114. 10.3390/ijms241210114 PMC10299208 37373261

[23] Tanabe Y, Nomura N, Minami M, Takaya J, Okamoto N, Yanagi K, et al. HIST1H1E syndrome with deficiency in multiple pituitary hormones. Clin Pediatr Endocrinol. 2023;32(3):195–8. 10.1297/cpe.2023-0002 PMC10288291 37362168

[24] Yuen KCJ, Johannsson G, Ho KKY, Miller BS, Bergada I, Rogol AD. Diagnosis and testing for growth hormone deficiency across the ages: a global view of the accuracy, caveats, and cut-offs for diagnosis. Endocr Connect. 2023;12:7. 10.1530/EC-22-0504 PMC10305501 37052176

[25] Stróżewska W, Durda-Masny M, Szwed AJG. Mutations in GHR and IGF1R Genes as a potential reason for the lack of catch-up growth in SGA children. Genes. 2022;13(5):856. 10.3390/genes13050856 PMC9140854 35627241

[26] Gui W, Liang J, Lin X, Shi N, Zhu Y, Tan B, et al. Association of genetic variants in IGF2-related genes with risk of metabolic syndrome in the chinese han population. Front Endocrinol. 2021;12:654747. 10.3389/fendo.2021.654747 PMC8173176 34093434

[27] Abdellatif M, Madeo F, Kroemer G, Sedej S. Spermidine overrides INSR (insulin receptor)-IGF1R (insulin-like growth factor 1 receptor)-mediated inhibition of autophagy in the aging heart. Autophagy. 2022;18(10):2500–2. 10.1080/15548627.2022.2095835 PMC9542397 35786404

[28] Fang K, Sun M, Leng Z, Chu Y, Zhao Z, Li Z, et al. Targeting IGF1R signaling enhances the sensitivity of cisplatin by inhibiting proline and arginine metabolism in oesophageal squamous cell carcinoma under hypoxia. J Exp Clin Cancer Res. 2023;42(1):73. 10.1186/s13046-023-02623-2 PMC10044411 36978187

[29] Clatot J, Parthasarathy S, Cohen S, McKee JL, Massey S, Somarowthu A, et al. SCN1A gain-of-function mutation causing an early onset epileptic encephalopathy. Epilepsia. 2023;64(5):1318–30. 10.1111/epi.17444 PMC10130239 36287100

[30] Kozycki CT, Kodati S, Huryn L, Wang H, Warner BM, Jani P, et al. Gain-of-function mutations in ALPK1 cause an NF-Î°B-mediated autoinflammatory disease: functional assessment, clinical phenotyping and disease course of patients with ROSAH syndrome. Ann Rheum Dis. 2022;81(10):1453–64. 10.1136/annrheumdis-2022-222629 PMC9484401 35868845

[31] Leiding JW, Vogel TP, Santarlas VGJ, Mhaskar R, Smith MR, Carisey A, et al. Monogenic early-onset lymphoproliferation and autoimmunity: Natural history of STAT3 gain-of-function syndrome. J Allergy Clin Immunol. 2023;151(4):1081–95. 10.1016/j.jaci.2022.09.002 PMC10081938 36228738

[32] Roos D, de Boer M. Mutations in cis that affect mRNA synthesis, processing and translation. Biochim Biophys Acta Mol Basis Dis. 2021;1867(9):166166. 10.1016/j.bbadis.2021.166166 33971252

[33] Zakaria NA, Bahar R, Abdullah WZ, Mohamed Yusoff AA, Shamsuddin S, Abdul Wahab R, et al. Genetic manipulation strategies for β-thalassemia: a review. Front Pediatr. 2022;10:901605. 10.3389/fped.2022.901605 PMC9240386 35783328

[34] Sharma M, Leung D, Momenilandi M, Jones LCW, Pacillo L, James AE, et al. Human germline heterozygous gain-of-function STAT6 variants cause severe allergic disease. J Exp Med. 2023;220(5):e20221755. 10.1084/jem.20221755 PMC10037107 36884218

[35] Wang L, Mirabella VR, Dai R, Su X, Xu R, Jadali A, et al. Analyses of the autism-associated neuroligin-3 R451C mutation in human neurons reveal a gain-of-function synaptic mechanism. Mol Psychiatry. 2022;27:1–16. 10.1038/s41380-022-01834-x PMC10123180 36280753

